# GATA4 regulates the transcription of *MMP9* to suppress the invasion and migration of breast cancer cells via HDAC1-mediated p65 deacetylation

**DOI:** 10.1038/s41419-024-06656-z

**Published:** 2024-04-23

**Authors:** Yuxi Yang, Shuangshuang Song, Shujing Li, Jie Kang, Yulin Li, Nannan Zhao, Dongman Ye, Fengying Qin, Yixin Du, Jing Sun, Tao Yu, Huijian Wu

**Affiliations:** 1 School of Bioengineering & Key Laboratory of Protein Modification and Disease, Liaoning Province, Dalian University of Technology, Dalian, 116024 China; 2 Cancer Hospital of Dalian University of Technology, Shenyang, 110042 China

**Keywords:** Breast cancer, Extracellular matrix

## Abstract

GATA-binding protein 4 (GATA4) is recognized for its significant roles in embryogenesis and various cancers. Through bioinformatics and clinical data, it appears that GATA4 plays a role in breast cancer development. Yet, the specific roles and mechanisms of GATA4 in breast cancer progression remain elusive. In this study, we identify GATA4 as a tumor suppressor in the invasion and migration of breast cancer. Functionally, GATA4 significantly reduces the transcription of *MMP9*. On a mechanistic level, GATA4 diminishes *MMP9* transcription by interacting with p65 at the NF-κB binding site on the *MMP9* promoter. Additionally, GATA4 promotes the recruitment of HDAC1, amplifying the bond between p65 and HDAC1. This leads to decreased acetylation of p65, thus inhibiting p65’s transcriptional activity on the *MMP9* promoter. Moreover, GATA4 hampers the metastasis of breast cancer in *vivo* mouse model. In summary, our research unveils a novel mechanism wherein GATA4 curtails breast cancer cell metastasis by downregulating *MMP9* expression, suggesting a potential therapeutic avenue for breast cancer metastasis.

## Introduction

Breast cancer is a predominant health risk for women globally, with alarming incidence and mortality rates [1]. The diverse nature of metastasis in breast cancer primarily contributes to its grim prognosis [2]. Notably, post primary tumor treatment, 20-30% of breast cancer patients experience metastasis [3], with a 5-year overall survival rate exceeding 80% for patients without metastasis [4]. However, tumor metastasis is a multi-step process with epithelial-to-mesenchymal transition (EMT) being pivotal in initiating cancer cell migration [5]. Due to existing diagnostic and therapeutic limitations, many breast cancer patients in the early metastatic stages do not receive optimal treatment.

Numerous reports suggest GATA4’s pivotal involvement in various cancer biological processes, including apoptosis, proliferation, and metastasis [6–8]. GATA4, belongs to the GATA transcription factor family. It possesses the distinct zinc-finger DNA binding domains that specifically recognize or bind the GATA response element (A/T) GATA (A/G) on gene promoters [9]. Concurrently, GATA4’s role in regulating ERα-mediated transcription highlights a potential link with breast cancer progression [10]. Supporting this, immunohistochemistry has shown that GATA4 status serves as a potential prognostic indicator for patient survival, pointing to GATA4’s possible influence on breast cancer progression [11]. Nevertheless, the molecular mechanisms detailing GATA4’s effect on breast cancer metastasis remain largely uncharted.

Typically, the microenvironment of tumors comprises the extracellular matrix (ECM), basement membrane, and vessels [12]. As a pivotal stage in cancer metastasis, a fundamental step of EMT is the degradation of the ECM by matrix metalloproteinases (MMPs). This process enables cancer cells to breach the basement membrane, promoting tumor cell invasion and movement [13]. Type IV collagen is the mainstay of the ECM [14, 15].

Among the MMPs, MMP2 and MMP9 stand out for their role in degrading type IV collagen. Specifically, the expression and activity of MMP9 hold significant relevance to breast cancer metastasis and tumor malignancy [16–18], and elevated MMP9 levels increased the malignancy of breast tumor [19, 20]. The region that close to the transcription-start-site of the *MMP9* encompasses vital gene regulatory response elements, including transcription factors AP-1, NF-κB, Sp1 and ETS-1. It’s established that the NF-κB response element oversees *MMP9*’s regulation via p65 and other transcriptional mediators [21]. Notably, the proximal AP-1 binding site collaborates with the NF-κB site to optimize *MMP9* expression [22]. In this context, NF-κB’s role is paramount in *MMP9* expression, with *MMP9* also being a direct downstream target of NF-κB [18].

There’s evidence to suggest that the NF-κB pathway is an activated oncogenic pathway in both breast cancer cells and tumor tissues [23] and is intricately linked to facilitate breast cancer progression, including proliferation, apoptosis, metastasis and inflammation [24]. P65, which belongs to the NF-κB transcription factor family, forms p65/p50 complex that carries out transcriptional activation functions to modulate downstream genes and is considered an oncogenic factor in breast cancer [25, 26]. This pathway governs a myriad of target genes, such as *c-Myc*, *VEGF*, and *MMPs* [27]. Additionally, NF-κB-mediated transcriptional activity can be further modulated by other transcription regulators, either amplifying or dampening its effects [28, 29]. Furthermore, post-translational modifications, like the acetylation of p65, play a role in guiding NF-κB-mediated transcriptional actions [30, 31]. Hence, the influence of NF-κB signaling in cancer progression is both broad and multifaceted.

In our research, we revealed that GATA4 inhibits the invasion and migration of breast cancer cells by downregulating *MMP9* expression. We’ve outlined a potential mechanism wherein GATA4 inhibits the transcriptional activation of p65 on the *MMP9* promoter via interaction with p65. Moreover, GATA4 seems to curtail p65’s transcriptional activity by strengthening the bond between p65 and HDAC1, leading to reduced acetylation of p65. Our insights into the unique molecular interplay of the GATA4/NF-κB/*MMP9* pathway shed new light on potential therapeutic avenues for breast cancer.

## Materials and methods

### Plasmids and cells

The plasmids were previously described [21], as follows: p*MMP9* luciferase reporter construct, truncated p*MMP9* luciferase reporter construct, mutant p*MMP9* luciferase reporter construct, Flag-p65, GFP-p65, Myc-HDAC1, HA-p300. Flag-GATA4 was kindly provided from Dr. Baohua Liu (Shenzhen University Health Science Center); HA-HDAC1 was kindly provided from Dr. Tieshan Tang (State Key Laboratory of Membrane Biology, Institute of Zoology, Chinese Academy of Sciences); Myc-GATA4-△ZF1, Myc-GATA4-△ZF2, and Myc-GATA4-△ZF1&△ZF2 were kindly provided form Dr. Liang Chen (Guangdong Province Key Laboratory of Bioengineering Medicine, Jinan University). Sh-GATA4 and GFP-GATA4 were constructed from our lab. The GST-p65 and GST-GATA4 were amplified by PCR and cloned into pGEX-4T-3 vector. The primers about the amplification and RNAi were listed in supplementary Table 1. T47D, MCF7, ZR-75-1, BT474, MCF10A, MDA-MB-231, HEK293T, 4T1 cells were previously described [32]; HCC1187 was cultured in Roswell Park Memorial Institute (RPMI) -1640 supplemented with 10% (v/v) fetal bovine serum (FBS); SKBR-3 was cultured in Dulbecco’s Modified Eagle Medium (DMEM) supplemented with 10% FBS; MDA-MB-453 was cultured in Leibovitz’s L-15 Medium supplemented with 10% FBS; cells were grown at 37 °C in a humidified 5% CO_2_ atmosphere.

### Transfection, antibodies and chemicals

HEK293T cells were transfected with polyethylenimine (PEI); MCF7, T47D, HCC1187 and MDA-MB-231 cells were transfected with Lipofectamine 3000 (Invitrogen, Auckland, New Zealand). Antibodies were used in our research were described in supplementary Table 2. TNF-α (a potent NF-kB pathway activator) was purchased from Selleck (Selleck, Houston, TX, USA) and used at final concentrations of 5 μM for 1 h; Isoginkgetin (a specific MMP9 inhibitor) was purchased from Selleck (Selleck, Houston, TX, USA) and used at final concentrations of 10 μM for 24 h.

### Western blot, immunoprecipitation, GST pull-down and immunofluorescence

Western blot, immunoprecipitation, GST pull-down and immunofluorescence assays were previously described [32]. For western blot and immunoprecipitation, total cellular proteins were extracted by TNE lysis buffer (Tris-HCl pH 7.4 20 mM, NaCl 100 mM, EDTA 1 mM, NP-40 0.5%, Glycerol and complete protease inhibitor 10%); Flag-affinity magnetic beads (P2115) were purchased from BeyoGold (Beyotime, Shanghai, China), and Protein A/G magnetic beads (B23201) were purchased from Selleck (Selleck, Houston, TX, USA). For GST-pull down, fusion proteins with GST-tag were expressed in E. coli *BL21*; GST-GATA4 and GST-p65 were induced with 0.08 mM Isopropyl β-D-Thiogalactoside IPTG (Solaribo, I8070) for 4 h at 30 °C while shaking; total cellular proteins were extracted by GST lysis buffer (NaCl pH 7.3 140 mM, KCl 2.7 mM, Na_2_HPO_4_ 10 mM, KH_2_PO_4_ 1.8 mM) and the next assay was described in BeyoGold GST-tag Purification Resin (BeyoGold, P2250).

### Cell scratch wound-healing and transwell assay

For scratch wound-healing assay, cells were transfected with indicated plasmids and plated in 6-well plate: 1 × 10^5^ cells per well in medium with 2% FBS for avoiding the effects of cell proliferation. For transwell assay, cells transfected with indicated plasmids were plated in transwell apical chamber, and the transwell apical chambers for invasion evaluation were pre-packaged with matrigel: 1 × 10^4^ cells per chamber in medium without FBS for invasion and migration. Next assays were previous described [32].

### Luciferase reporter assay

Cells were plated into a 24-well plate, and transfected with indicated plasmids; 24 h later, cells were lysed by lysis buffer: (pH 7.8, Glycine 2.5 mM, EGTA 4 mM, MgSO_4_ 15 mM, DTT 1 mM, TritonX -100 1%). The luciferase activity assay was measured by Centro LB 960 and described previously [21].

### Gelatin zymography

Gelatin zymography assay was performed as previously described [21]. Cells were transfected indicated plasmids and incubated in medium with 10% FBS; after 24 h, cells were washed with PBS and incubated in serum-free medium for 24 h. Supernatants were collected and electrophoresed on 10% SDS-polyacrylamide gel containing 1% gelatin. According to MMP Zymography Assay Kit (GEMIC, XF-P17750), the gel was stained and distained after incubation.

### Chromatin immunoprecipitation

Chromatin immunoprecipitation (ChIP) assay was performed as previously described [33]. Cells were transfected with indicated plasmids and plated in 100 mm plate. The primers used in the ChIP PCR analysis for *MMP9* promoter were listed in supplementary Table 1.

### Mice metastasis model

Mice metastasis model was performed as previously described [21]. Female BALB/C mice (5–6 weeks old, 16–18 g) were purchased from Liaoning Changsheng Biotechnology and maintained under specific pathogen-free (SPF) conditions. Mice were divided into three groups randomly. Each group of mice were implanted with 100 μl PBS buffer, or 4T1 cells (1 × 10^6^ cells in 100 μl PBS buffer), or GATA4 transfected-4T1 cells (1 × 10^6^ cells in 100 μl PBS buffer) by tail intravenous injection respectively. Mice weights of each group were recorded every day after injection. After 10 days, mice were euthanized with CO_2_, and their lungs were harvested for weighing and imaging purposes and stored in formalin or liquid nitrogen for subsequent analysis. All animal experiments were approved by the Ethics Committee for Biology and Medical Science of Dalian University of Technology.

### Quantification of lung metastasis in mice

Hematoxylin and eosin (H&E) staining of mice metastasis lungs was performed as previously described [34]. Histopathological analysis was performed using ImageJ software to determine the percentage of metastatic area to total area in lungs; the number of metastatic nodules was counted in the lungs of the mice.

### RNA isolation, RT-PCR and qPCR

Total RNA was extracted by Takara RNAiso Reagent (Takara, 9109). The PrimeScript™ RT Master Mix (Takara, RR036A) was used for RT-PCR (Reverse Transcription). The SYBR® Premix Ex Taq™ II (Takara, RR820A) was used for qPCR (quantitative Real-time). The mRNA levels were measured by ABI Prism 7500 sequence detection system. The specific primers for real-time PCR were listed in supplementary Table 3.

### Statistical analysis

GraphPad Prism 9 software was used for data analysis, and data were showed as means ± SDs from at least three independent experiments. An unpaired *t*-test was used when the results from two groups were compared. Experimental data is considered to be significant when *p* < 0.05. *p < 0.05, **p < 0.01, ***p < 0.001.

## Results

### GATA4 inhibits the invasion and migration of breast cancer cells

**Fig. 1:**
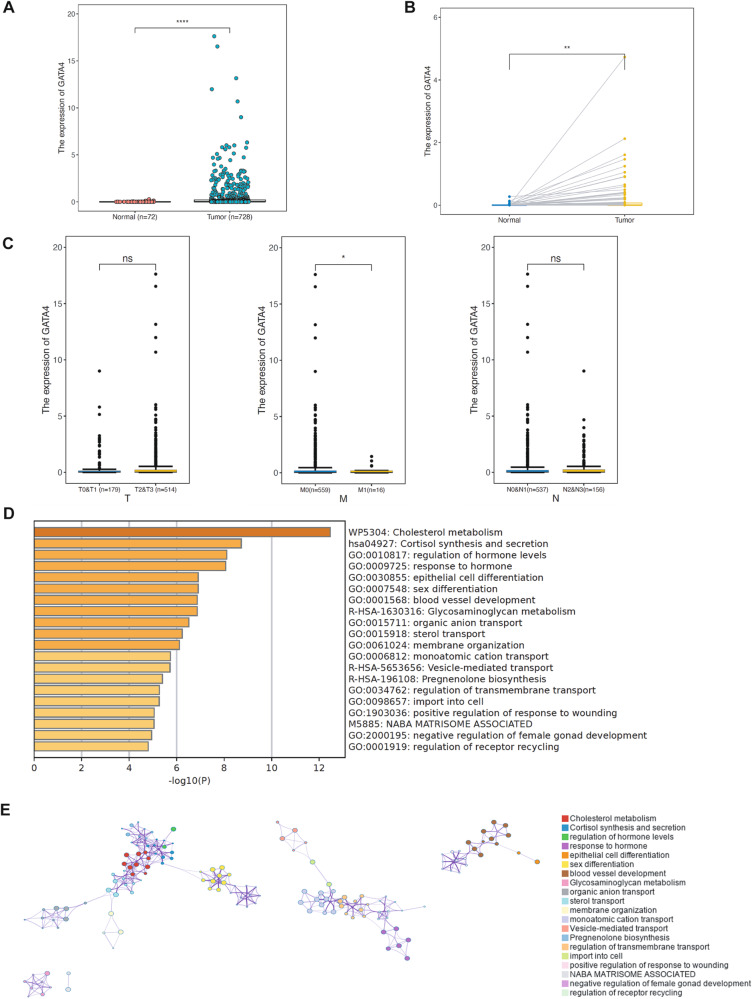
Bioinformatics analysis of GATA4 and associated genes in breast cancer. **A** GATA4 expression of adjacent normal tissues in breast cancer. (The gene expression data and clinical data of breast cancer patient were obtained from TCGA database and analyzed with R Studio. Normal: *n* = 72; Tumor: *n* = 728). **B** GATA4 expression of paired adjacent normal tissues in breast cancer. (The data of 72 breast cancer cases with tumor and normal tissues were obtained from TCGA database and analyzed with R Studio. Normal: *n* = 72; Tumor: *n* = 72). **C** GATA4 expression in different TMN (Tumor-Metastasis-Node) stages in breast cancer. (The gene expression data of breast cancer patient with TMN stage data were obtained from TCGA database and analyzed with R Studio). **D**, **E** GO enrichment analysis (**D**) and cluster analysis (**E**) of biological processes of GATA4 positively associated genes (Pearson Correlation Coefficient > 0.1) in breast cancer by Metascape (http://metascape.org/). (NABA MATRISOME ASSOCIATED: Nidogen-1 and 2, Agrin, Perlecan, and Basement membrane components extracellular matrix).

Existing clinical data indicate that GATA4 seems to exhibit association with breast cancer progression [11, 35], though the exact molecular mechanism remains to be defined. Our initial step was to evaluate the pan-cancer expression of GATA4 using TIMER2.0. This analysis showed that GATA4 expression was elevated in certain tumors, including breast cancer, prostate adenocarcinoma, and thyroid carcinoma, compared to their adjacent normal tissues (Fig. S1A). Diving deeper into breast cancer, our analysis revealed that GATA4 mRNA levels were notably higher in breast carcinoma tissues compared to normal tissues and similarly in breast cancer paired tissues (Fig. 1A, B). The specific role of GATA4 in tumorigenesis and progression was still controversial. Therefore, we further analyzed whether the expression of GATA4 changes along with the progression of Tumor-Metastasis-Node stages in breast cancer. Notably, GATA4 expression was substantially reduced in stage M1 compared to M0, suggesting lower GATA4 expression in cancer tissues with metastasis (Fig. 1C). Moreover, the variation of GATA4 expression among breast cancer subtypes was analyzed by UALCAN, and this analysis showed that GATA4 expression tends to be lower in subtypes with more malignant character (Fig. S1B).

**Fig. 2:**
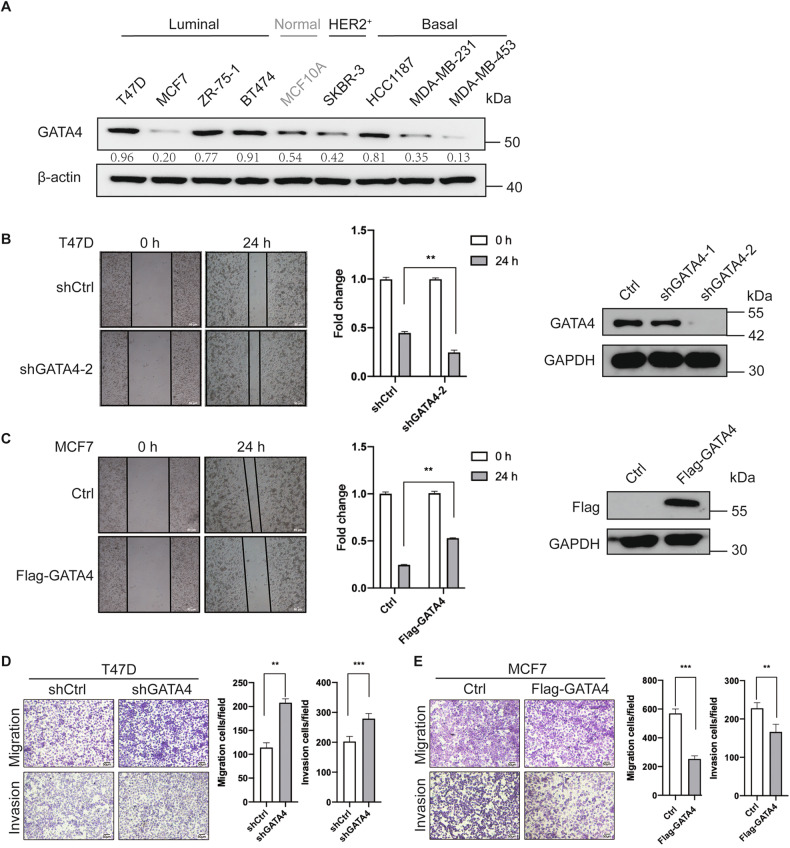
GATA4 inhibits breast cancer cells motility, invasion. **A** GATA4 expression in normal breast cell and different types of breast cancer cells. (The relative expression of GATA4 was normalized by β-actin with gray level analysis by ImageJ). **B**, **C** Scratch wound-healing assay showed the effect of GATA4 knockdown in T47D cells (**B**) and overexpression in MCF7 cells (**C**), data are the means ± SDs from three determinations. **p < 0.01; western blot tested the effect of GATA4 knockdown with shRNAs and overexpression with Flag-GATA4. **D**, **E** Transwell assay showed the effect of GATA4 knockdown in T47D cells (**D**) and overexpression in MCF7 cells (**E**), data are the means ± SDs from three determinations. **p < 0.01; ***p < 0.001.

To better understand GATA4’s role in metastasis, we undertook a biological process enrichment analysis via Metascape. The genes associated with GATA4 (Table S4) played a part in regulating EMT, cell motility (positive regulation of response to wounding) and ECM (NABA MATRISOME ASSOCIATED) pathway. Since these processes are pivotal to cancer cell metastasis, it points to the potential influence of GATA4 on the EMT by possibly modulating the ECM (Fig. 1D, E). The expression of GATA4 did not have a significant correlation with the survival of breast cancer patients as expected (Fig. S1C), so we speculated that GATA4 is a potential metastatic marker in breast cancer. Collectively, this suggests that GATA4 might function as a metastatic regulator in breast cancer. To further delve into this hypothesis, we firstly assessed GATA4’s expression in various breast cancer cell lines. We observed that GATA4 expression appeared lower in cell lines with mesenchymal or aggressive characters (except HCC1187) and higher in cell lines with epithelial character (except MCF7) (Fig. 2A). To pinpoint GATA4’s function in breast cancer metastasis, we conducted cell scratch wound-healing and transwell assays in MCF7 and T47D cells or MDA-MB-231 and HCC1187 cells to eliminate possible effects by different breast cancer subtypes. Our findings showed that silencing GATA4 augmented the migratory and invasive capabilities of the cancer cells (Fig. 2B and D, Fig. S2B, D and F). On the flip side, cells with GATA4 expression displayed decreased migration and invasion tendencies (Fig. 2C and E, Fig. S2A, C and E). Consequently, our data support the idea that GATA4 acts as a suppressor of breast cancer cell migration and invasion.

### GATA4 decreases the expression of *MMP9* in breast cancer cells

**Fig. 3:**
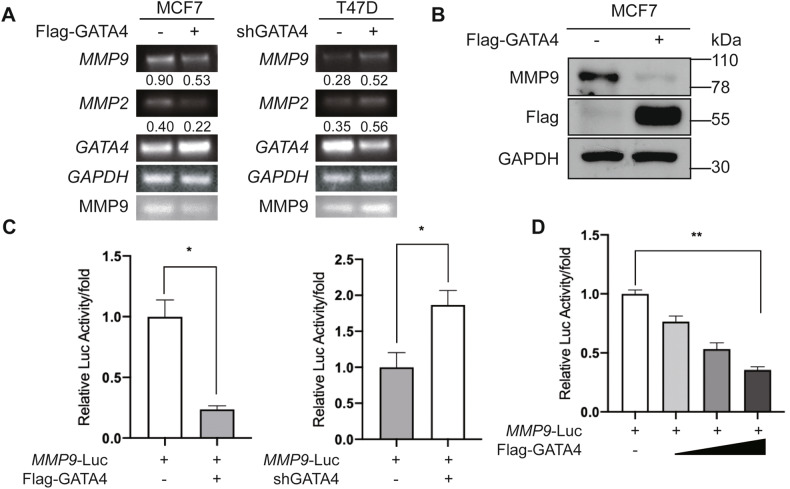
GATA4 inhibits the expression of MMP9 in breast cancer cells. **A** PCR assay showed the effect of GATA4 knockdown in T47D cells and overexpression in MCF7 cells on the mRNA expression of *MMP2* and *MMP9*; the medium from cells was collected for gelatin zymography (the last line). (The relative expression of *MMP9* and *MMP2* was normalized by *GAPDH*). **B** Western blot assay showed the effect of GATA4 overexpression in MCF7 cells on the protein expression of MMP9. **C** The luciferase reporter assay showed the effect of GATA4 overexpression and knockdown in HEK293T cells on *MMP9* promoter. Data are the means ± SDs from three determinations. *p < 0.05. **D** The luciferase reporter assay showed the effect of GATA4 overexpression (0 ng, 200 ng, 400 ng, 600 ng) in HEK293T cells on *MMP9* promoter. Data are the means ± SDs from three determinations. **p < 0.01.

Prior research highlighted GATA4’s potential to reduce MMP2 expression in breast cancer cells [6]. Given that MMP2 and MMP9 play important roles in degrading type IV collagen with MMP9 being particularly influential in breast cancer metastasis, we sought to determine if GATA4 might also counteract breast cancer metastasis by modulating MMP9. Our findings indicated that augmenting GATA4 expression led to a decline in *MMP9* mRNA levels, while inhibiting GATA4 increased *MMP9* expression (Fig. 3A). Consistent with earlier studies, our data also revealed that GATA4 inhibited *MMP2* expression (Fig. 3A). Furthermore, elevating GATA4 levels reduced MMP9 protein amounts (Fig. 3B). To discern whether GATA4 could transcriptionally govern *MMP9* expression, we employed luciferase reporter assays to assess GATA4’s impact on *MMP9*’s promoter. Our observations demonstrated that amplifying GATA4 diminished the activity of *MMP9*-Luc. Conversely, suppressing GATA4 bolstered the activity of *MMP9*-Luc (Fig. 3C). Intriguingly, GATA4 appeared to curtail *MMP9*-Luc activity in a dose-responsive manner (Fig. 3D). This collective evidence suggests that GATA4 likely suppresses *MMP9* expression at the transcriptional stage.

### Determination of GATA4 binding elements on *MMP9* promoter

**Fig. 4:**
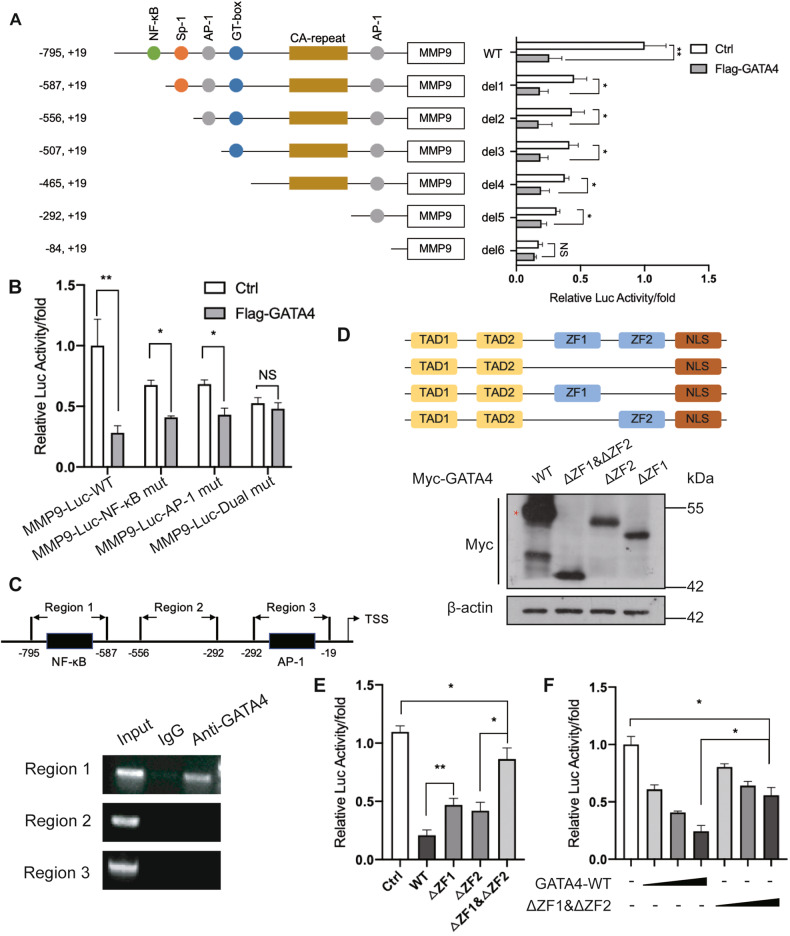
Determination of GATA4 binding sites on *MMP9* promoter. **A** HEK293T cells were respectively co-transfected with a series of 5’- deletion constructs of human *MMP9* promoter reporter plasmids, and either Flag-GATA4 or control vector for 24 h, and luciferase activities were measured. Data are the means ± SDs from three determinations. *p < 0.05; **p < 0.01; ns, no significance. **B** HEK293T cells were respectively co-transfected with wild-type and mutant of human *MMP9* promoter (mutant of NF-κB response element and AP-1 response element) reporter plasmids, and either Flag-GATA4 or control vector for 24 h, and luciferase activities were measured. Data are the means ± SDs from three determinations. *p < 0.05; **p < 0.01; ns, no significance. **C** Chromatin immunoprecipitation assay showed the binding region of GATA4 on *MMP9* promoter. Top, schematic illustration of PCR-amplified fragments of *MMP9* promoter; bottom, ChIP assay was screened for Flag-GATA4-bound *MMP9* promoter regions in HEK293T cells. **D** Western blot assay showed the expression of GATA4 truncated constitutions in HEK293T cells. Top, schematic illustration of truncated constitutions of GATA4; bottom, the expression of GATA4 truncated constitutions. **E** The luciferase reporter assay showed the effect of GATA4 truncated constitutions in HEK293T cells on *MMP9* promoter. Data are the means ± SDs from three determinations. *p < 0.05; **p < 0.01. **F** The luciferase reporter assay showed the effect of GATA4 and its truncated constitutions (0 ng, 200 ng, 400 ng, 600 ng) in HEK293T cells on *MMP9* promoter. Data are the means ± SDs from three determinations. *p < 0.05.

To delve deeper into the mechanism by which GATA4 suppresses *MMP9* expression, we used a luciferase reporter linked to truncated versions of the *MMP9* promoter (Fig. 4A, left). As shown in Fig. 4A, the full-length *MMP9* promoter displayed the most pronounced inhibition by GATA4. This inhibitory effect waned when the promoter was shortened to -587 bp, implying GATA4’s influence on the NF-κB response element situated between -795 bp and -587 bp. Further shortening to -84 bp further reduced GATA4’s inhibitory impact, suggesting a potential influence on the AP-1 response element found between -292 bp and -84 bp. Given that the *MMP9* promoter lacks conventional GATA4 binding sites, it seems plausible that GATA4 serves more as a transcriptional modulator rather than a direct transcription factor for *MMP9*. Subsequent experiments utilized mutated luciferase reporters linked to the *MMP9* promoter: NF-κB-mut-Luc, AP-1-mut-Luc, and dual-mut-Luc (Fig. S3A). Both individual mutations showed repression by GATA4, with the dual mutation revealing even stronger repression (Fig. 4B). This confirms GATA4’s inhibitory via both NF-κB and AP-1 elements of the *MMP9* promoter. Further pinpointing GATA4’s active regions on the *MMP9* promoter, we employed chromatin immunoprecipitation (ChIP) and identified a predominant enrichment of GATA4 in the NF-κB response element of *MMP9* promoter, with no noticeable association in the other two regions (Fig. 4C). These findings suggest that GATA4 likely constrains *MMP9* expression predominantly via the NF-κB response element but not AP-1, utilizing both direct and indirect regulatory strategies. GATA4 is characterized by two distinct zinc-finger domains: zinc-finger 1 (ZF1) and zinc-finger 2 (ZF2), which facilitate its interactions with DNA or proteins [9, 36]. Comparing the effects of the full-length GATA4 and versions lacking the zinc-finger domains on *MMP9* showed that while the intact GATA4 had the most potent repressive effect on the *MMP9* promoter, omitting either ZF1 or ZF2 tempered this suppression (Fig. 4D, E). When both ZF1 and ZF2 were excluded, GATA4’s repression significantly diminished, and increasing the expression of this truncated GATA4 didn’t restore the inhibitory effect (Fig. 4E, F). This highlights that GATA4’s transcriptional regulation of *MMP9* largely depends on ZF1 and ZF2 domains. In summary, GATA4 dampens the expression of *MMP9* on the transcriptional level by binding directly to the NF-κB response element and exerting an indirect effect on the AP-1 element.

### GATA4 interacts with p65

**Fig. 5:**
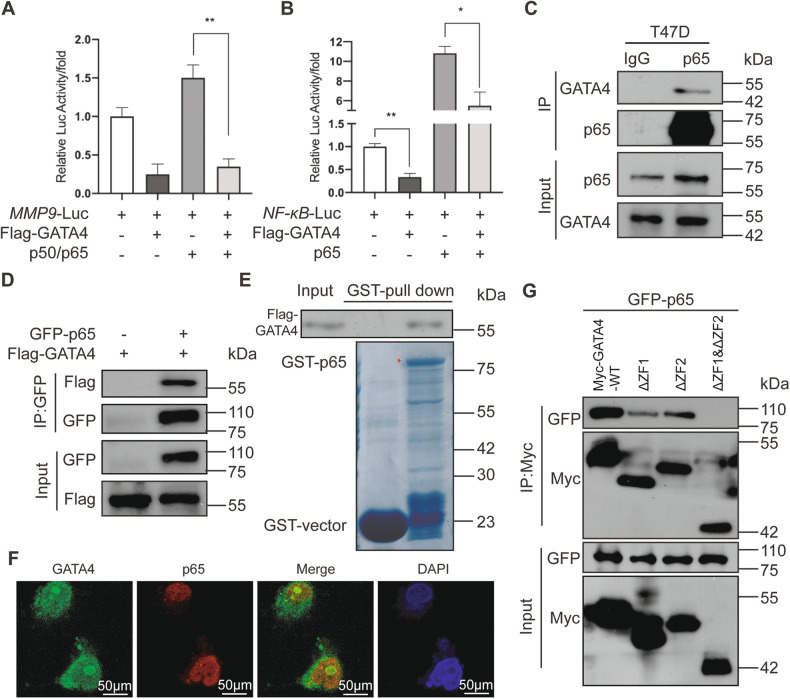
The interaction between p65 and GATA4. **A** The luciferase reporter assay showed the effect of p50/p65 in HEK293T cells on *MMP9* promoter with or without GATA4. Data are the means ± SDs from three determinations. **p < 0.01. **B** The luciferase reporter assay showed the effect of p65 in HEK293T cells on NF-κB response element of *MMP9* promoter with or without GATA4. Data are the means ± SDs from three determinations. *p < 0.05; **p < 0.01. **C** Lysates in T47D cells were immunoprecitated using anti-p65 Ab. Purified cell extracts were tested by western blot with anti-GATA4 Ab. **D** HEK293T cells were co-transfected with GFP-p65 and Flag-GATA4, and lysates were immunoprecitated using anti-GFP Ab. Purified cell extracts were tested by western blot with anti-Flag Ab. **E** GST-pulldown assay showed the purified GST-p65 in E. coli *BL21* had physical interaction with Flag-GATA4 in HEK293T cells. **F** T47D cells were stained with an anti-GATA4 (green) and an anti-p65 (red). Nuclei were stained with DAPI (blue), followed by visualization with confocal microscopy. **G** HEK293T cells were co-transfected with Myc-GATA4 or GATA4 truncated constitutions with GFP-p65, and lysates were immunoprecitated using anti-Myc Ab. Purified cell extracts were tested by western blot with anti-GFP Ab.

To investigate the potential interplay between GATA4 and NF-κB pathway in *MMP9* regulation, particularly given the absence of a GATA4-binding motif on the *MMP9* promoter, we hypothesized that GATA4 might work in tandem with other transcription factors at the NF-κB response element of the *MMP9* promoter. Within the NF-κB signaling, p65/p50 is known to directly bind to the *MMP9* promoter’s NF-κB response element, playing an essential role in controlling *MMP9* expression in breast cancer [37]. Our luciferase reporter assays revealed that p65/p50 overexpression significantly enhanced the activity of *MMP9*-Luc. Interestingly, the combined addition of both GATA4 and p65/p50 had a profoundly suppressive effect on this activity (Fig. 5A). This observation was further buttressed by findings showing the GATA4-mediated repression of the *MMP9* promoter via p65 was contingent upon the NF-κB response element (Fig. 5B). These results underscore the notion that GATA4 can counteract the transcriptional activation driven by p65/p50, leading to a reduction in *MMP9* expression. To dissect the potential interaction between GATA4 and p65, we embarked on co-immunoprecipitation experiments. Notably, both endogenous and overexpressed GATA4 displayed robust interaction with p65 (Fig. 5C, D). The GST-pull-down added another layer of evidence by confirming a direct, physical interaction between GATA4 and p65 (Fig. 5E). Additionally, through immunofluorescence, we visualized the co-localization of GATA4 and p65 within T47D cells (Fig. 5F and S3B). The interaction between GATA4 and p65 may be modulated by GATA4’s structural components. Deletion experiments demonstrated that while removing either the ZF1 or ZF2 domain from GATA4 attenuated its interaction with p65, the interaction was completely obliterated when both zinc-finger domains were absent (Fig. 5G). Since the transcriptional inhibition of GATA4 on *MMP9* promoter relies on the interaction with p65, GATA4 without the zinc-finger domains does not have significant inhibitory effects. This establishes that the synergy between GATA4 and p65 is contingent on the integrity of GATA4’s zinc-finger domains. In summation, our findings elucidate a novel interplay in breast cancer cells, where GATA4 curtails the influence of the p65/p50 complex on *MMP9* expression by interacting with p65.

### GATA4 facilitates HDAC1-mediated deacetylation of p65

**Fig. 6:**
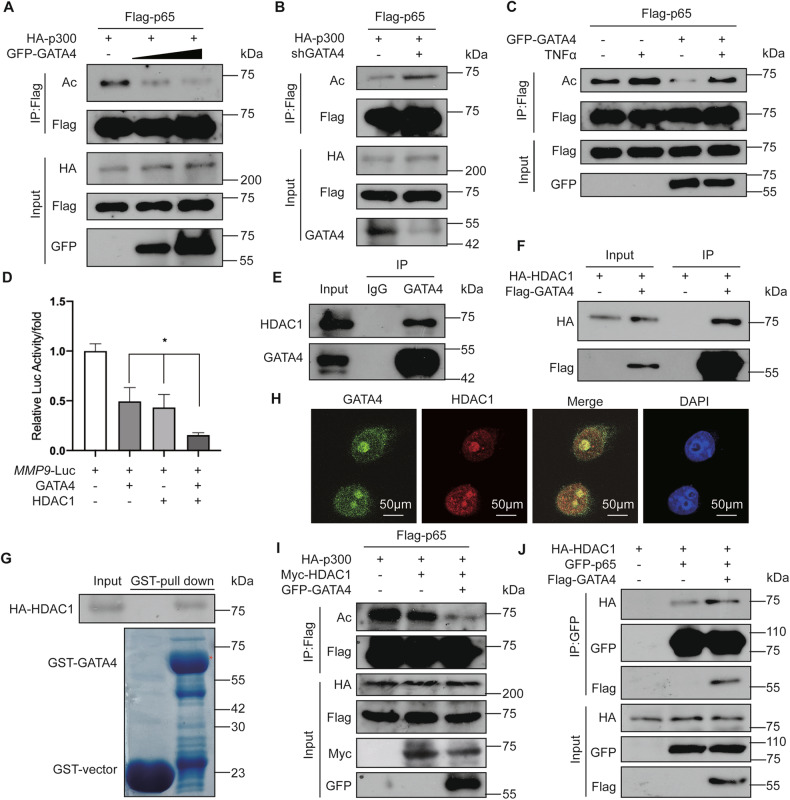
GATA4 facilitates HDAC1-mediated deacetylation of p65 by enhancing the interaction between p65 and HDAC1. **A**, **B** HEK293T cells were transfected with indicated plasmids, followed by immunoprecipitation with anti-Flag affinity magnetic beads and western blot with anti-AcK Ab. **C** HEK293T cells were transfected with indicated plasmids, and incubated with or without TNFα (5 μM for 1 h), followed by immunoprecipitation with anti-Flag affinity magnetic beads and western blot with anti-AcK Ab. **D** The luciferase reporter assay showed the effect of HDAC1 in HEK293T cells on *MMP9* promoter with or without GATA4. Data are the means ± SDs from three determinations. *p < 0.05. **E** Lysates in T47D cells were immunoprecitated using anti-GATA4 Ab. Purified cell extracts were tested by western blot with anti-HDAC1 Ab. **F** HEK293T cells were co-transfected with HA-HDAC1 and Flag-GATA4, and lysates were immunoprecitated using anti-Flag affinity magnetic beads. Purified cell extracts were tested by western blot with anti-HA Ab. **G** GST-pulldown assay showed the purified GST-GATA4 in E. coli *BL21* had physical interaction with HA-HDAC1 in HEK293T cells. **H** T47D cells were stained with an anti-GATA4 Ab (green) and an anti-HDAC1 Ab (red). Nuclei were stained with DAPI (blue), followed by visualization with confocal microscopy. **I** HEK293T cells were transfected with indicated plasmids, followed by immunoprecipitation with anti-Flag affinity magnetic beads and western blot with anti-AcK Ab. **J** HEK293T cells were co-transfected with HA-HDAC1, GFP-p65, and Flag-GATA4, and lysates were immunoprecitated using anti-GFP Ab. Purified cell extracts were tested by western blot with anti-HA and anti-Flag Ab.

Transcriptional activation mediated by NF-κB can be modulated by various factors to either amplify or suppress its effects [28]. Given that p65’s influence on transcription largely hinges on its acetylation [30], we assessed the acetylation of p65 in the presence of added GATA4 using western blot. Enhanced expression of GATA4 was observed to inhibit p65 acetylation (Fig. 6A), whereas reducing GATA4 amplified the acetylation of p65 (Fig. 6B). Notably, when treated with TNFα, GATA4’s inhibitory impact on p65 acetylation was still pronounced, suggesting GATA4’s potential to suppress p65 acetylation, especially when the NF-κB pathway is active (Fig. 6C). This infers that GATA4 might advance the deacetylation of p65, thereby repressing *MMP9* transcription. Previous research has indicated that TNFα diminishes HDAC1’s association with NF-κB binding sites on the *MMP9* promoter, leading to increased *MMP9* expression. HDAC1 has also been noted to engage with p65, downregulating gene expression via p65 deacetylation [38, 39]. Luciferase reporter assays demonstrated that amplifying either GATA4 or HDAC1 markedly suppressed *MMP9*-Luc activity, with a combined elevation of GATA4 and HDAC1 producing an even stronger inhibitory outcome (Fig. 6D). Moreover, interactions between GATA4 and HDAC1 were both endogenously and exogenously confirmed (Fig. 6E, F). To ascertain a direct interaction between GATA4 and HDAC1, we utilized a GST-pull-down assay involving GST-GATA4 and HA-HDAC1. Our findings illustrated that GATA4 can directly interact with HDAC1 (Fig. 6G). Immunofluorescence assays further verified the co-localization of GATA4 and HDAC1 in T47D cells (Fig. 6H and S3C). In addition, relative to the overexpression of HDAC1 alone, a diminished acetylation pattern of p65 was evident when both HDAC1 and GATA4 were jointly overexpressed (Fig. 6I). Recognizing GATA4’s capacity to decrease p65 acetylation led us to investigate if GATA4 might influence the liaison between p65 and HDAC1. Co-immunoprecipitation indicated a more robust interaction between HDAC1 and p65 in GATA4’s presence compared to its absence (Fig. 6J). Considering that GATA4-mediated repression of the *MMP9* promoter via p65 relied on the NF-κB response element (Fig. 5B), we also detected the downregulation of several NF-κB target genes mRNA levels (*VEGFA*, *TNFα*, and *uPA*) in MCF7 cells with GATA4 overexpression (Fig. S3D). This underscores GATA4’s role in bolstering the affinity between HDAC1 and p65. In sum, GATA4 appears to increase HDAC1’s recruitment to p65 and enhances HDAC1-driven deacetylation of p65, leading to a reduction in p65’s transcriptional activity on the *MMP9* promoter.

### GATA4 inhibits the metastasis of breast cancer cells

**Fig. 7:**
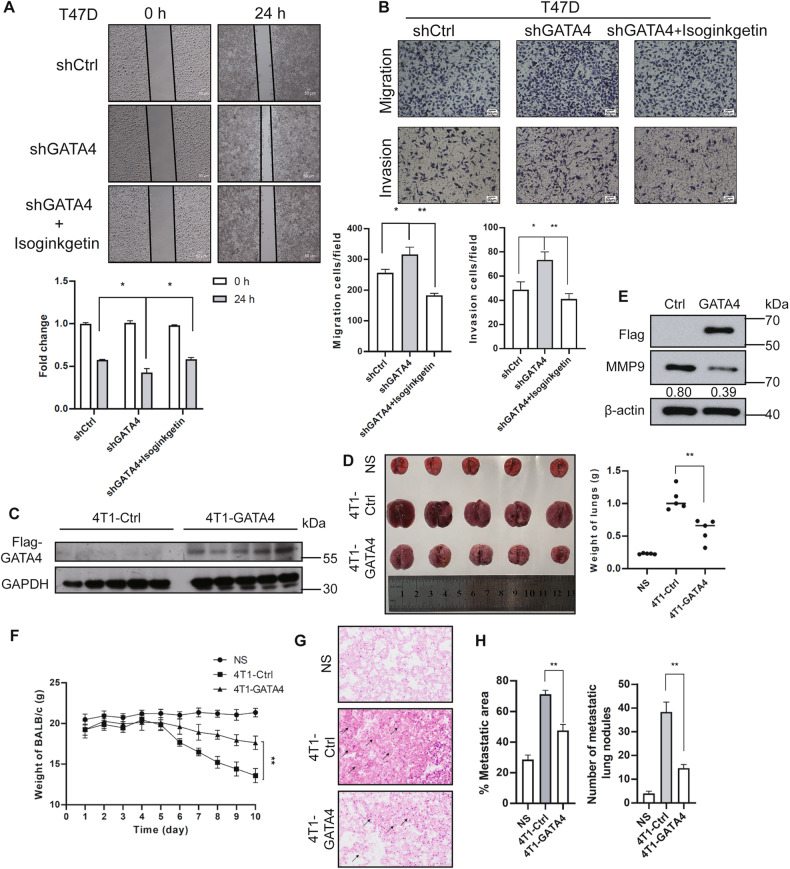
GATA4 inhibits the metastasis of breast cancer. **A** Scratch wound-healing assay showed the effect of GATA4 knockdown in T47D cells with or without MMP9 inhibitor (Isoginkgetin: 10 μM for 24 h). Bottom, data are the means ± SDs from three determinations. *p < 0.05. **B** Transwell assay showed the effect of GATA4 knockdown in T47D cells with or without MMP9 inhibitor (Isoginkgetin). Bottom, data are the means ± SDs from three determinations. *p < 0.05; **p < 0.01. **C** Western blot showed the Flag-GATA4 expression when overexpressed Flag-GATA4 into 4T1 cells. These cells were used to inject into BALB/C mice. **D** Image showed the lungs metastasis of BALB/C mice (left). Scatter plots image showed the lung weight of BALB/C mice (right). Data are the means ± SDs from five determinations. **p < 0.01. **E** Western blot analysis of the expression of Flag-GATA4 and MMP9 in 4T1-Ctrl and 4T1-GATA4 lungs. **F** The line graph shows the quantitative measure of the weight of mice. Data are the means ± SDs from five determinations. **p < 0.01. **G** H&E staining evaluating the lung metastasis of the three groups (NS; 4T1-Ctrl; and 4T1-GATA4). Arrows point to metastatic nodules. **H** Percent lung metastatic area in the three groups (left). Number of metastatic lung nodules in the three groups (right). Data are the means ± SDs from three determinations. **p < 0.01.

Previously, GATA4 was identified as a tumor suppressor in breast cancer [6]. Given the critical role of MMP9 in breast cancer metastasis and the inhibitory effect of GATA4 on *MMP9* transcription, we sought to further assess the influence of GATA4 on breast cancer metastasis. Utilizing cell scratch wound-healing and transwell assays, we observed that, relative to the control group, cells with GATA4 knockdown exhibited enhanced migration and invasion capabilities. Notably, the introduction of a specific MMP9 inhibitor (Isoginkgetin [21]) substantially mitigated the heightened migration and invasion triggered by the absence of GATA4 (Fig. 7A, B). This indicates that GATA4’s suppression of breast cancer cell metastasis operates chiefly through its regulation of *MMP9*. 4T1 cell line, which is highly tumorigenic and invasive, is considered as a suitable experimental model for breast cancer [40]. To delve into GATA4’s role in a live setting, 4T1 cells with stable GATA4 expression were administered to 5-week-old BALB/C mice (Fig. 7C). Cells producing GATA4 manifested reduced metastatic tendencies, as evidenced by the lung size and weight (Fig. 7D), mice’s weight (Fig. 7F), and lung metastatic-nodules (Fig. 7G, H). In contrast, cells containing the control vector were more proficient in fostering tumor metastasis. Examination of the expression level of Flag-GATA4 in the lungs showed that Flag-GATA4 was successfully expressed in the lungs and the level of MMP9 in the lungs was also down-regulated (Fig. 7E). In conjunction with our prior molecular mechanism analysis and validation, these findings compellingly argue for GATA4’s role in curtailing breast cancer metastasis.

## Discussion

While the GATA4 serves many roles in various cells and tissues, our study centered on investigating its potential as a specific therapeutic target for cancer. Our findings highlight GATA4’s inhibitory on *MMP9* and delve into its unique mechanism in counteracting breast cancer invasion and metastasis. By analyzing clinical breast cancer samples from TCGA, we discerned the downregulation of GATA4 in metastatic breast cancer tissues. Genes related to GATA4 predominantly function in cancer metastasis pathways (Fig. 1). Concurrently, we established that GATA4 impedes breast cancer cell invasion and metastasis (Fig. 2). Furthermore, we unveiled that GATA4 recruits HDAC1 to diminish p65 acetylation, leading to a suppression of *MMP9* transcription (Figs. 3–6). Mice model validated GATA4’s inhibitory stance on breast cancer cell metastasis (Fig. 7). This research accentuates GATA4’s role in curtailing breast cancer metastasis, offering fresh insights into GATA4’s tumor suppressive function during breast cancer progression, and underscores its potential as a therapeutic focal point for metastatic breast cancer. However, there are several limitations and issues in our study that we need to discuss and prospect.

Past studies identified GATA4’s inhibitory influence on hepatocellular carcinoma, colorectal cancer, and lung cancer via the modulation of pathways like Wnt/β-catenin [36], NOTCH/IRG1 [41] and TGFβ [42], while GATA4 acts as a cancer booster on acute lymphocytic leukemia by MDM2/p53 pathway. Our research revealed the inhibitory effect on breast cancer of GATA4 via NF-κB pathway. These findings indicate that the activation of the different signaling pathways is linked to different cancer cells, and this may be the reasons for different functions of GATA4 come into play. For another, the differential expression of GATA4 may also modulate cell differentiation. Earlier studies have intimated that GATA4 downregulates the expression of EMT markers in breast cancer cells, it’s plausible that GATA4 might be a significant inhibitor of EMT during breast cancer metastasis [6]. Especially as the expression of GATA4 in breast cancer inversely correlates with the adverse prognostic marker ERBB2 [35]. However, our analysis of breast cancer patients in the TCGA database showed that the expression level of GATA4, a tumor suppressor, was higher in carcinoma than in adjacent tissue, and this phenomenon was also observed in pancreatic cancer [8]. Previous studies have indicated that the mutation at the Y38 and P103 sites of GATA4 affect its transcriptional activity [43]. Based on these findings, we hypothesized that mutations in GATA4 within cancer tissues may account for its high expression yet inability to perform its tumor suppressor function, like the tumor suppressor protein p53 [44]. In addition, the SUMOylation of GATA4 at the K366 site has been shown to augment its transcriptional activity [45], suggesting that the SUMOylation of GATA4 could be another determinant of its tumor suppressive effect.

Some heterogeneous populations of cancer cells formed tumor cell heterogeneity in tumor tissues, and ECM is considered one of the important inducements for intratumor heterogeneity in the tumor microenvironment [46]. Our results showed that GATA4 expression appeared lower in cell lines with mesenchymal and aggressive characters (except HCC1187) and higher in cell lines with epithelial character (except MCF7), and repressed cell invasion and metastasis ability. We hypothesize that there are cell populations in breast tumors that are responsible for secreting MMPs, which set the stage for the invasion and metastasis of cancer cells with stronger cell mobility by changing the state of ECM in the tumor microenvironment, to promote the occurrence of metastatic breast cancer. Thus, we believe that the differential expression of GATA4 across various breast cancer cell lines, and its regulated expression of MMP9, could contribute to intratumor heterogeneity. In addition, we verified other NF-κB downstream genes (*VEGFA*, *TNFα*, and *uPA*) which involved in the regulation of tumor microenvironment and found that GATA4 can also down-regulate their mRNA levels, suggesting that GATA4 may be a potential factor in the regulation of tumor microenvironment.

GATA4 comprises two highly conserved zinc-finger domains, ZF1 and ZF2 [9]. ZF2 distinctively identifies and attaches to the (A/T) GATA (A/G) sequence, while ZF1 ensures stability in DNA-protein or protein-protein bindings [47]. Moreover, previous reports suggest that ZF1 and ZF2 facilitate GATA4’s protein interactions [36]. Our analysis revealed that both ZF1 and ZF2 curtail *MMP9* transcription by bolstering the GATA4-p65 interaction. However, the role of TAD and NLS domains in maintaining the GATA4-p65 interaction warrants further exploration. Our research asserted that GATA4 mitigates breast cancer cell metastasis through the downregulation of *MMP9* transcription. Upon examining the human *MMP9* promoter sequence, we pinpointed GATA4 as a potential regulator of NF-κB and AP-1 response elements. Notably, while the NF-κB response element emerged as a GATA4 binding site, AP-1 did not. The AP-1 response element, located ~70 bp upstream of the *MMP9* promoters, is believed to be vital for activating the transcription of *MMP9* [33]. Given that c-Jun, an AP-1 family member, has been known to synergize with GATA transcription factors on the GATA or AP-1 response elements [48], we speculate that GATA4’s indirect regulation of the *MMP9* promoter could be orchestrated by the c-Jun/AP-1 response element. Further studies are warranted to elucidate this potential indirect regulation of the AP-1 response element by GATA4.

A key mechanism through which the NF-κB pathway encourages tumor invasion and metastasis is by elevating *MMP9* expression [18]. Despite GATA4’s specific affinity for the (A/T) GATA (A/G) sequence, there isn’t a discernible GATA4-binding motif within the *MMP9* promoter. This led us to postulate that GATA4 might be ushered to the *MMP9* promoter via other molecules. Within the NF-κB signaling cascade, the p65/p50 duo attaches to the NF-κB response element on target gene promoters to stimulate transcription [27]. Our investigations revealed a direct interaction between GATA4 and p65, resulting in diminished *MMP9* expression. This suggests that GATA4 might modulate the transcriptional activity of the p65/p50 complex. Interestingly, removal of the ZF1 and ZF2 domains disrupted the GATA4-p65 connection, underscoring the importance of these domains for GATA4 to exert its influence on *MMP9* via p65. Transcriptional amplification steered by p65/p50 is open to further modulation by other transcription factors or post-translational modifications. Notable factors in this regulatory framework include coactivators like CBP/p300 [30], and repressors such as HDAC1, HDAC2, and HDAC3 [29, 49]. Upon activation by TNFα, the NF-κB pathway promotes p65 acetylation by facilitating the recruitment of p300 and simultaneously releasing HDAC1 [39]. In our study, we showcased that GATA4 bolsters the association between HDAC1 and p65, consequently reducing the acetylation level of p65 and curbing *MMP9* expression. Nevertheless, it’s crucial to acknowledge that GATA4 might also lure other NF-κB-linked repressors or vie with coactivators. Hence, the complete spectrum of ways GATA4 might influence the NF-κB pathway merits deeper exploration. While our research zoomed in on the impact of GATA4 on p65, owing to its role in the transcriptional activation of the p65/p50 complex, the dynamics between GATA4 and p50 remain an enigma, warranting further scrutiny.

GATA4’s influence extends to more than just *MMP9* in breast cancer. Prior research has highlighted GATA4’s capability to reduce MMP2 and MMP3 expression in breast cancer [6]. *MMP3*’s regulation by NF-κB mirrors that of *MMP9*: p65 can directly bind to the NF-κB response element on the *MMP3* promoter [50]. Likewise, *MMP1* and *MMP10* are also the downstream gene of p65 with NF-κB response element on the promoter [50, 51]. Given this resemblance, it’s plausible to suggest that GATA4 may regulate these *MMPs* expression akin to its modulation of *MMP9*. Yet, the relationship between NF-κB and *MMP2* is mediated through MT1-MMP [52]. Furthermore, studies have hinted that GATA4 might reduce p65 at the mRNA level. However, the intricacies of this mechanism remain veiled [6]. While GATA4’s diverse modulation of the NF-κB pathway is evident, it’s pivotal to bolster these observations with more concrete evidence.

**Fig. 8:**
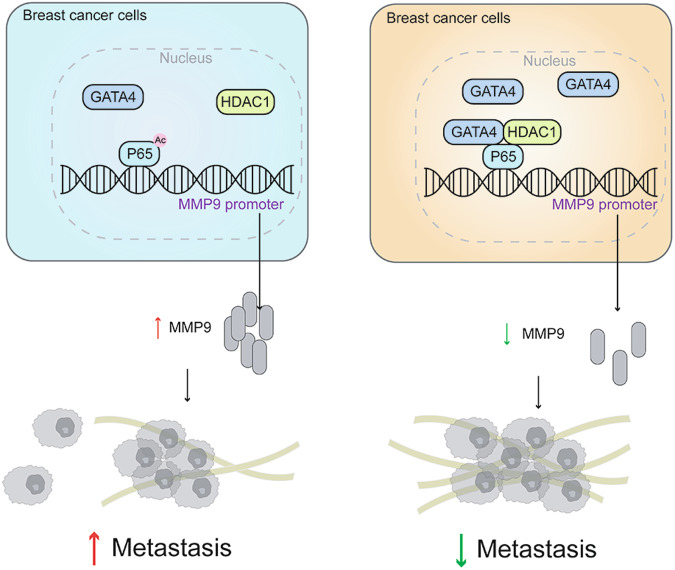
Graphic overview. A graphic overview depicts that GATA4/p65/HDAC1 axis inhibits the metastasis of breast cancer cells. The specific interaction between GATA4 and p65 weakened the activated role of p65 on the *MMP9* promoter by facilitating HDAC1-mediated deacetylation of p65.

In summary, our research has unearthed a probable mechanism wherein GATA4, in tandem with HDAC1, diminishes *MMP9* expression by curbing the acetylation of p65. Zooming in, we discerned that GATA4’s interaction with p65 curtails *MMP9* expression. Additionally, GATA4 recruits HDAC1 to the GATA4/p65 complex, leading to decreased acetylation of p65 (Fig. 8). This underpins GATA4’s role in thwarting breast cancer metastasis. Given these insights, the GATA4/p65/HDAC1 complex emerges as a promising therapeutic target for breast cancer.

### Reporting summary

Further information on research design is available in the Nature Research Reporting Summary linked to this article.

### Supplementary information

Supplementary Figure 1

Supplementary Figure 2

Supplementary Figure 3

Supplementary Table 1

Supplementary Table 2

Supplementary Table 3

Supplementary Table 4

Supplementary Figures and tables legends

Original WB data

Reporting Summary

## Data Availability

The data that support the findings of this study are available from the corresponding author upon request.
