# Supplementary figures and images for: GATA4 regulates the transcription of MMP9 to suppress the invasion and migration of breast cancer cells via HDAC1-mediated p65 deacetylation
Source: Cell Death Dis. 2024 Apr 23;15(4):289. doi: 10.1038/s41419-024-06656-z (PMC11039647; doi:10.1038/s41419-024-06656-z)

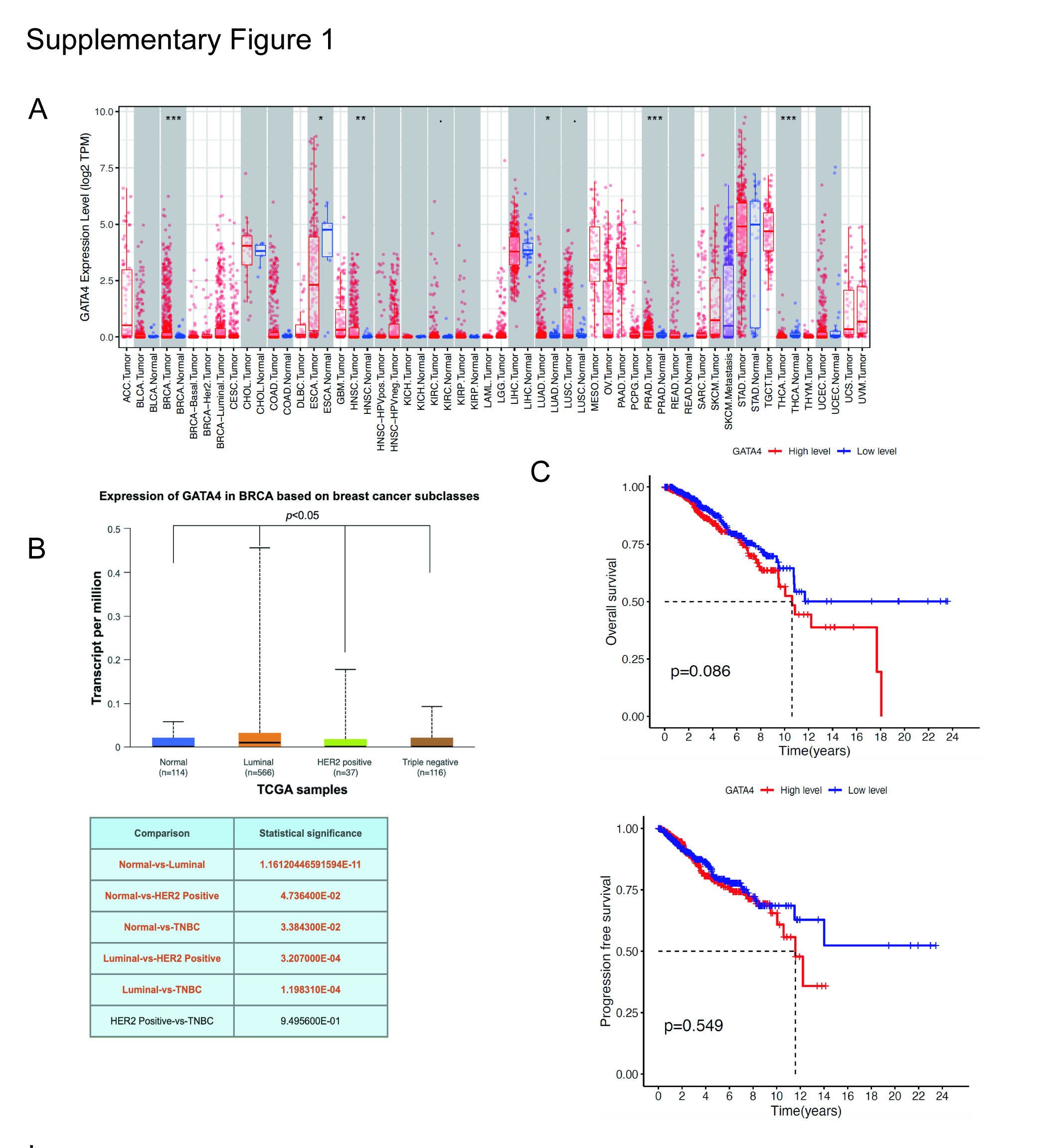

Supplement: Supplementary file 1 — Supplementary Figure 1 [file 41419_2024_6656_MOESM1_ESM.tif]

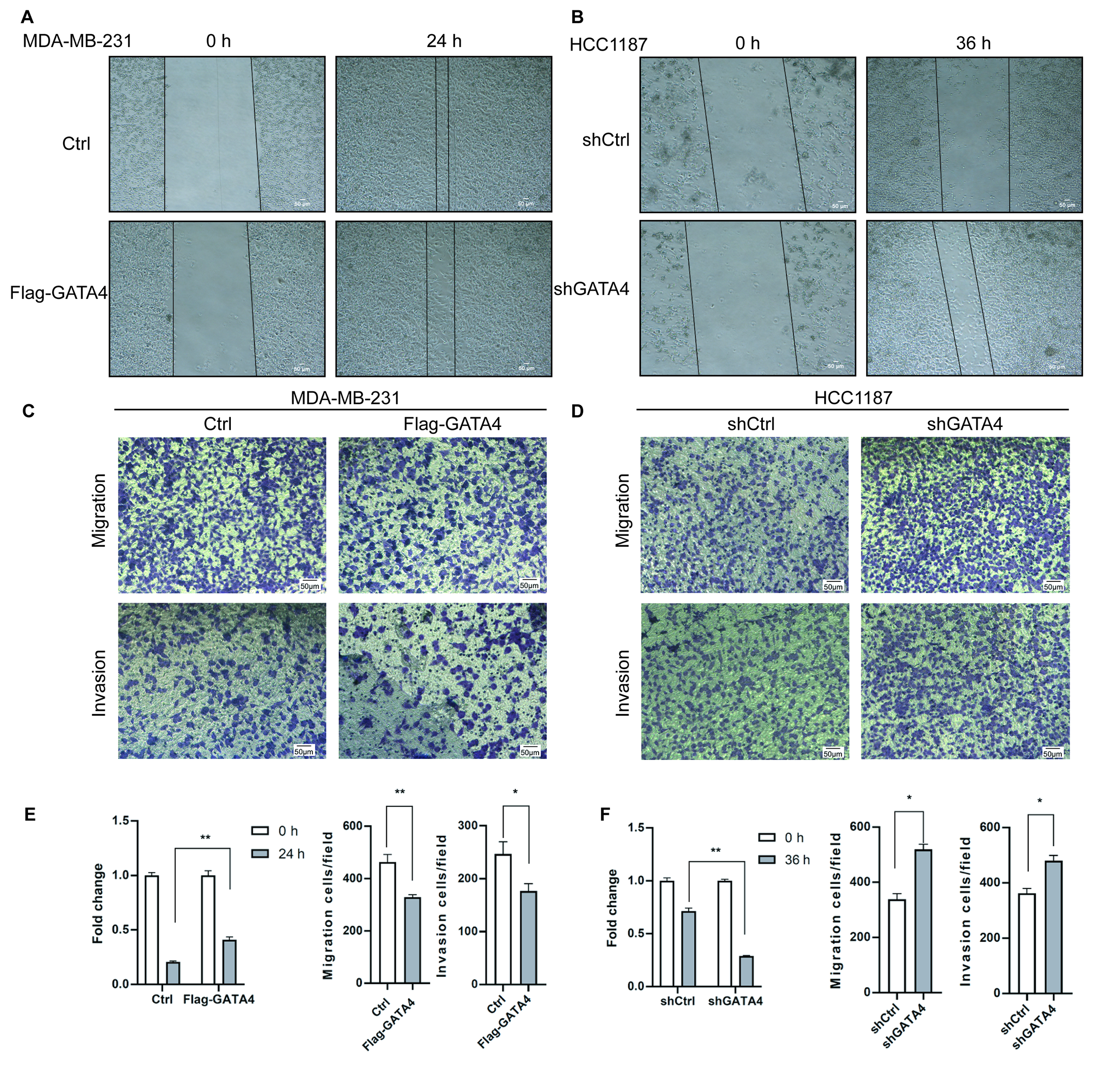

Supplement: Supplementary file 2 — Supplementary Figure 2 [file 41419_2024_6656_MOESM2_ESM.tif]

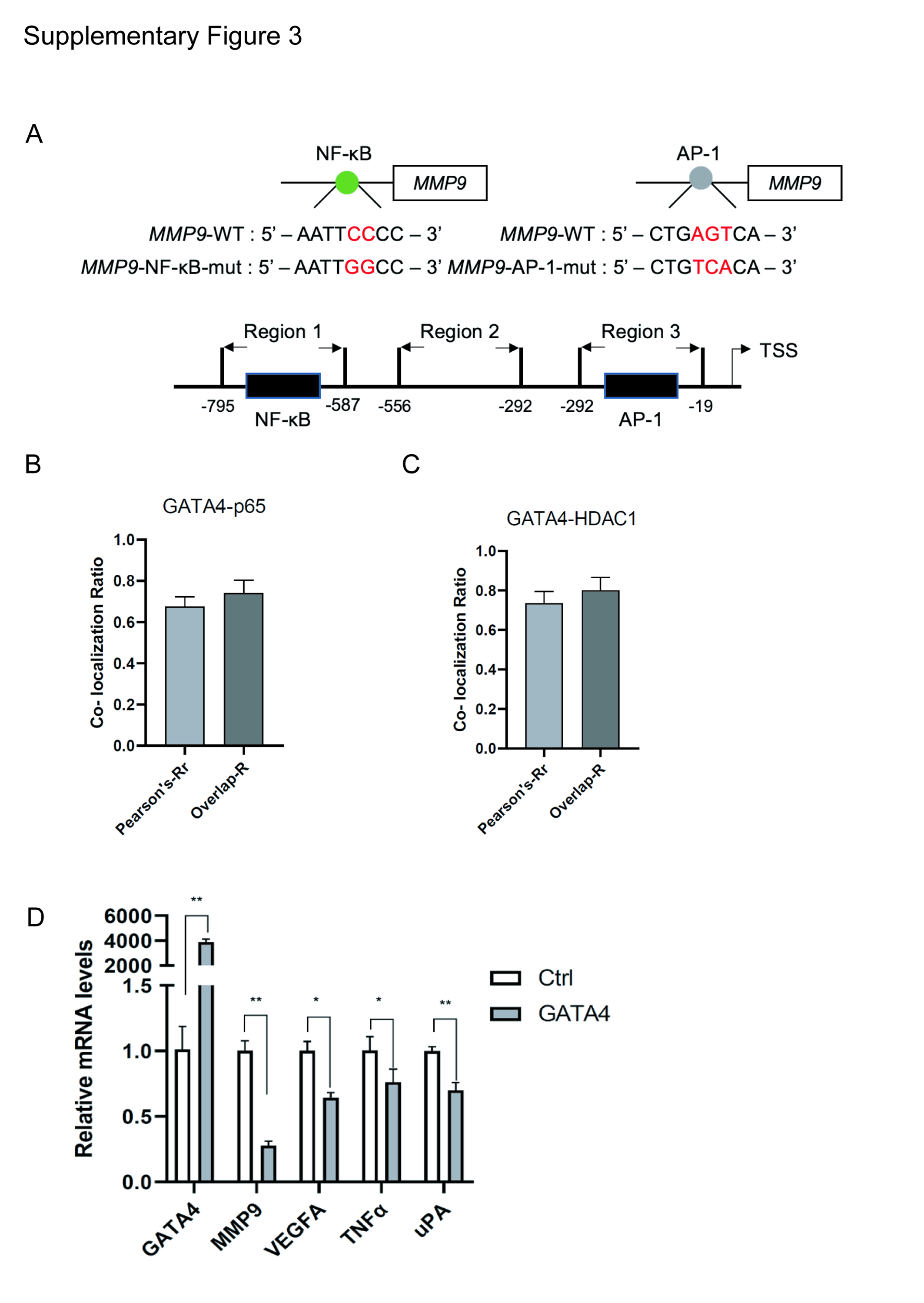

Supplement: Supplementary file 3 — Supplementary Figure 3 [file 41419_2024_6656_MOESM3_ESM.tif]

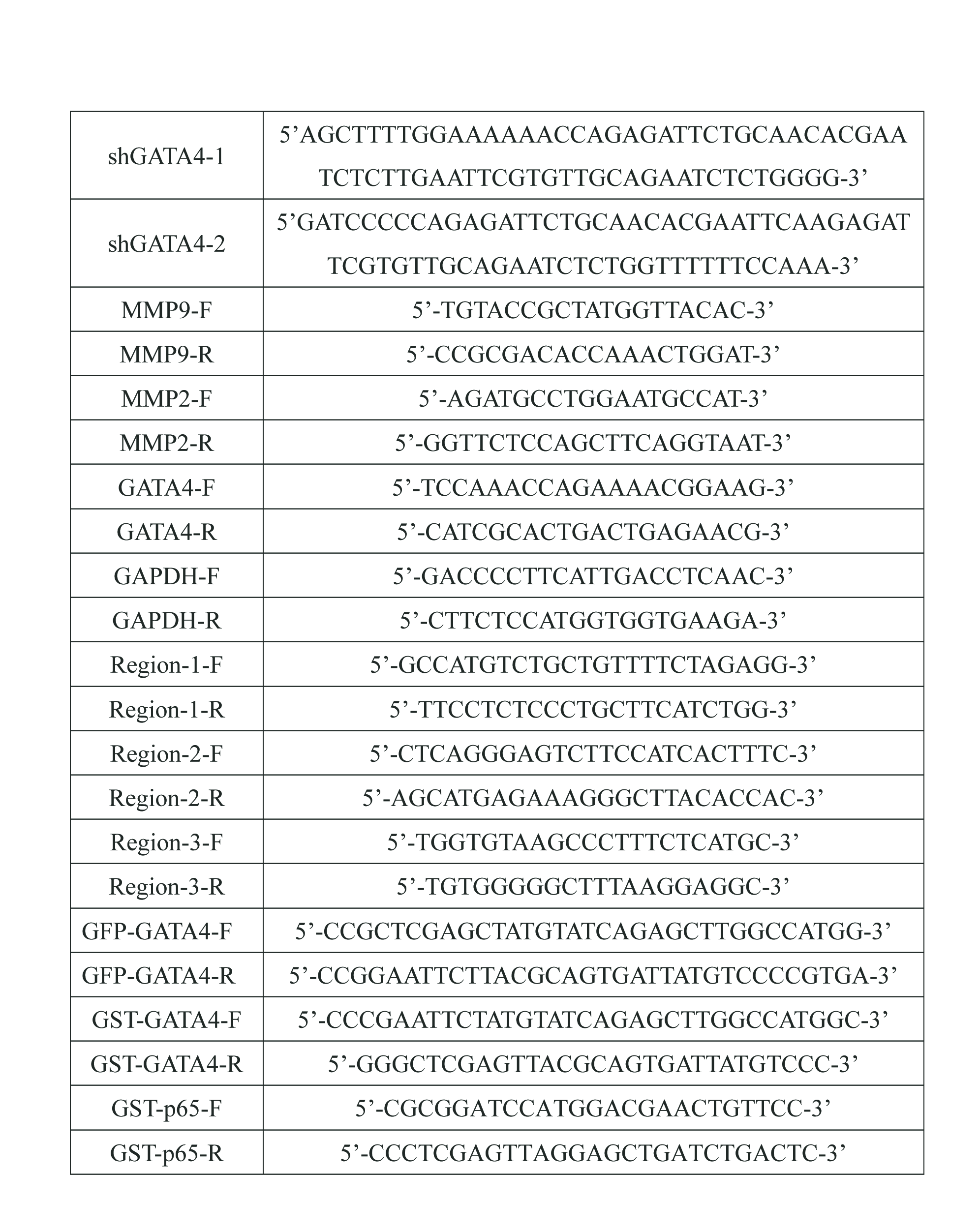

Supplement: Supplementary file 4 — Supplementary Table 1 [file 41419_2024_6656_MOESM4_ESM.tif]

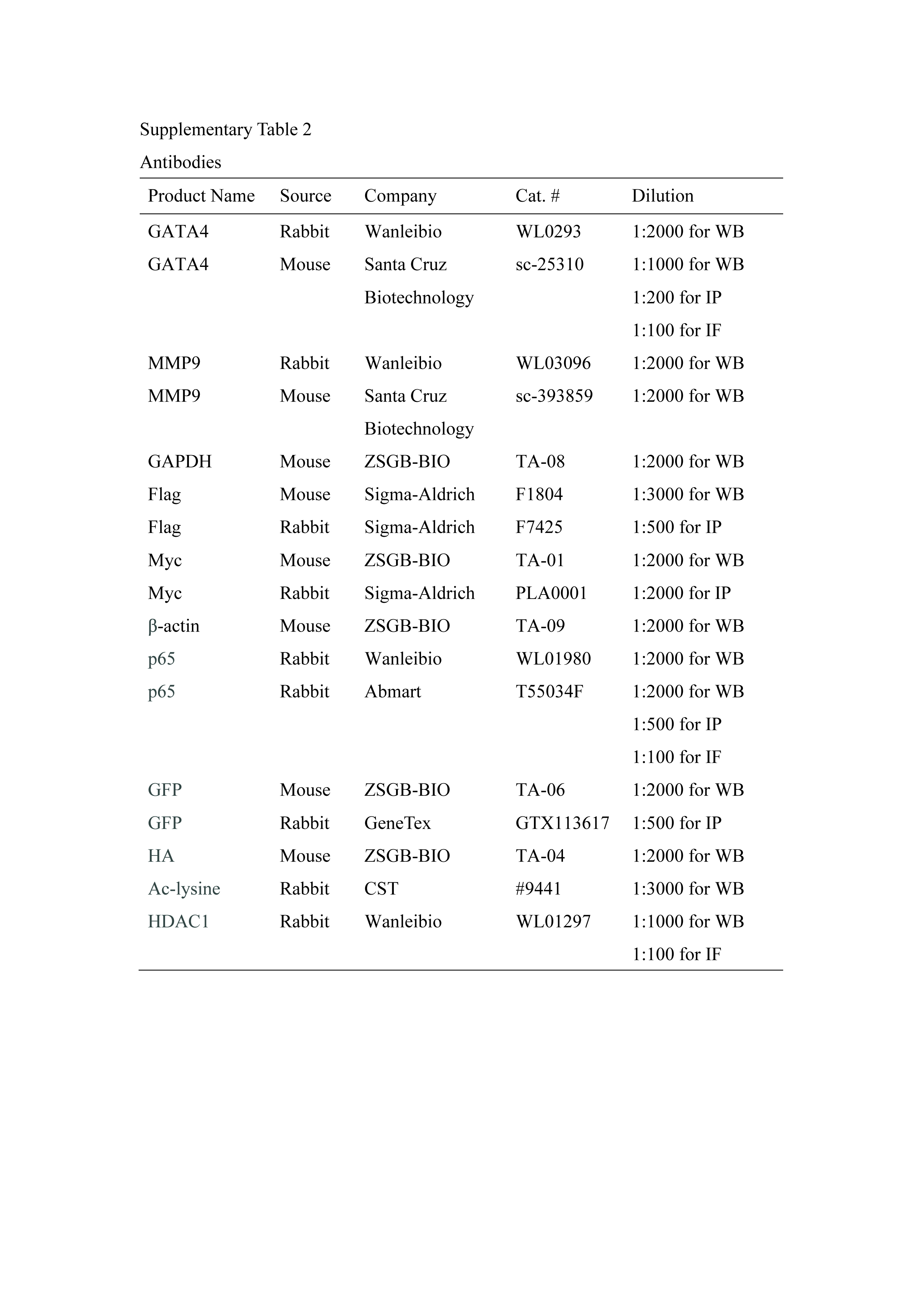

Supplement: Supplementary file 5 — Supplementary Table 2 [file 41419_2024_6656_MOESM5_ESM.tif]

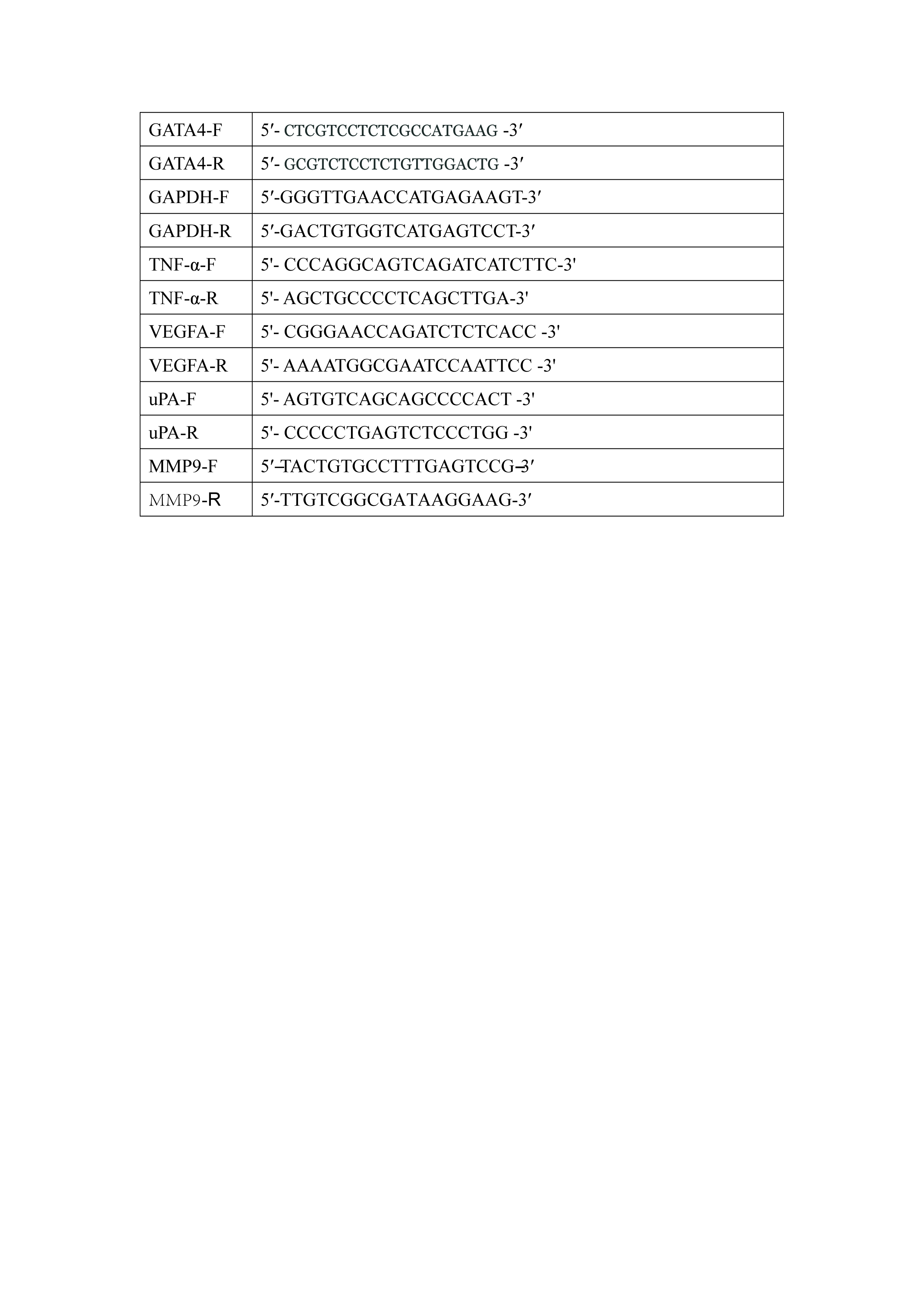

Supplement: Supplementary file 6 — Supplementary Table 3 [file 41419_2024_6656_MOESM6_ESM.tif]

Figure 2

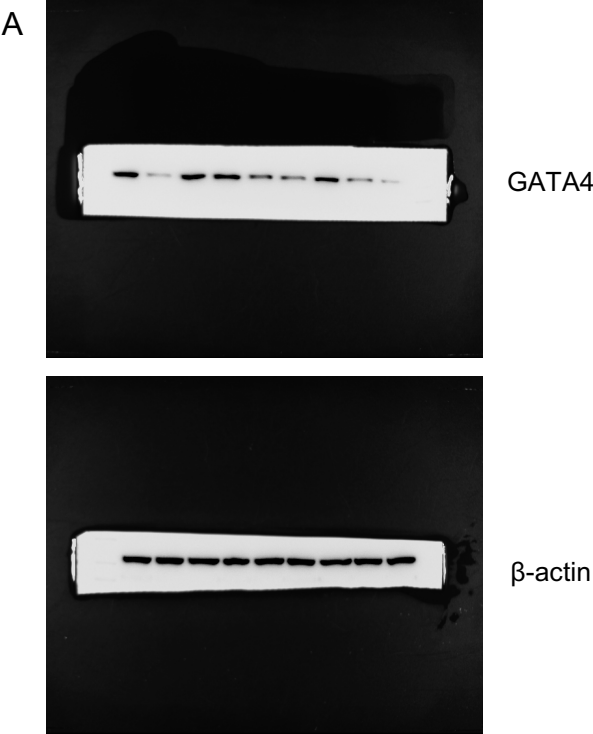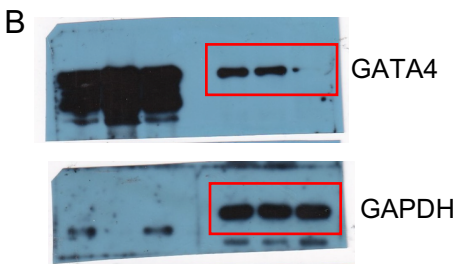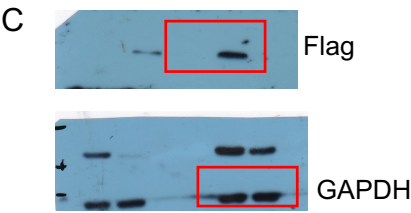

Figure 3

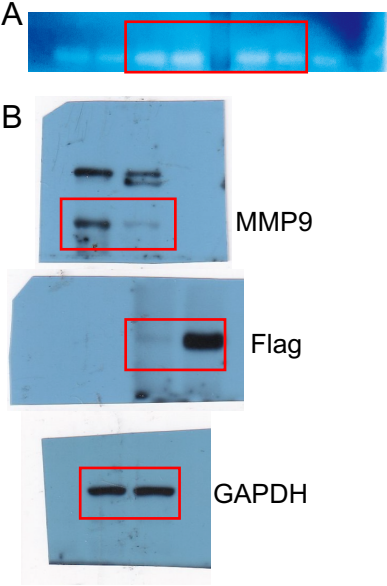

Figure 4

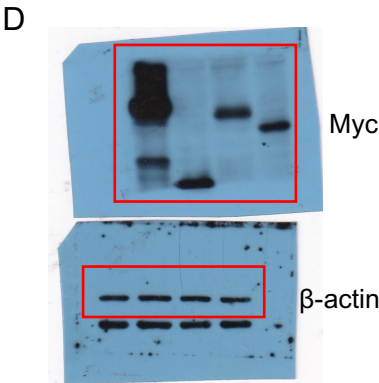

Figure 5

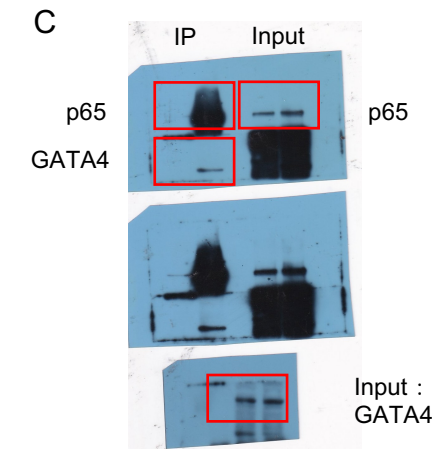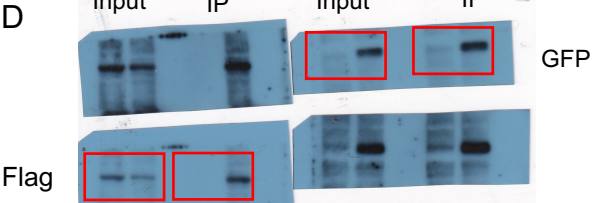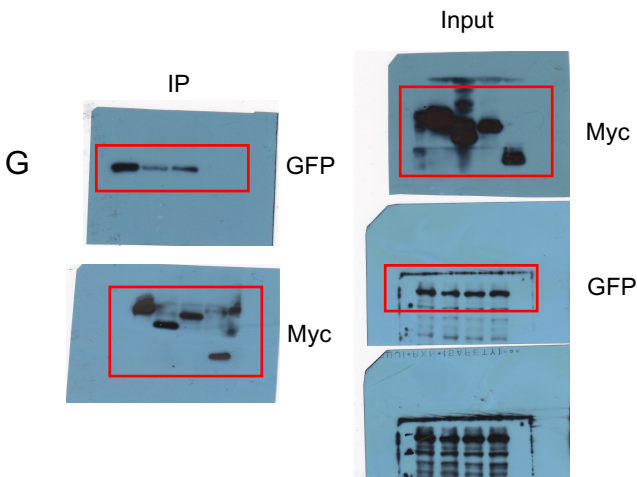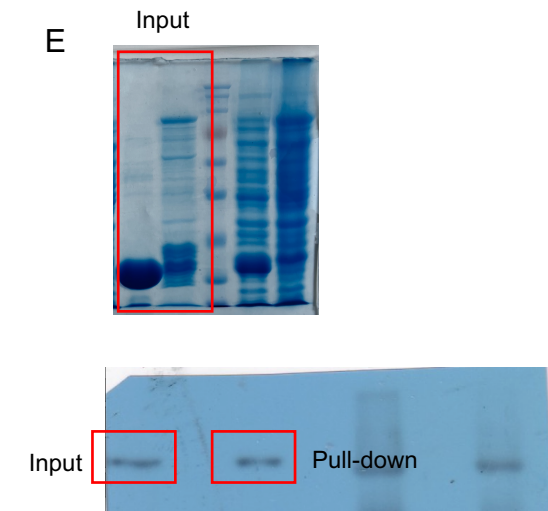

Figure 6

A (right), B (left)

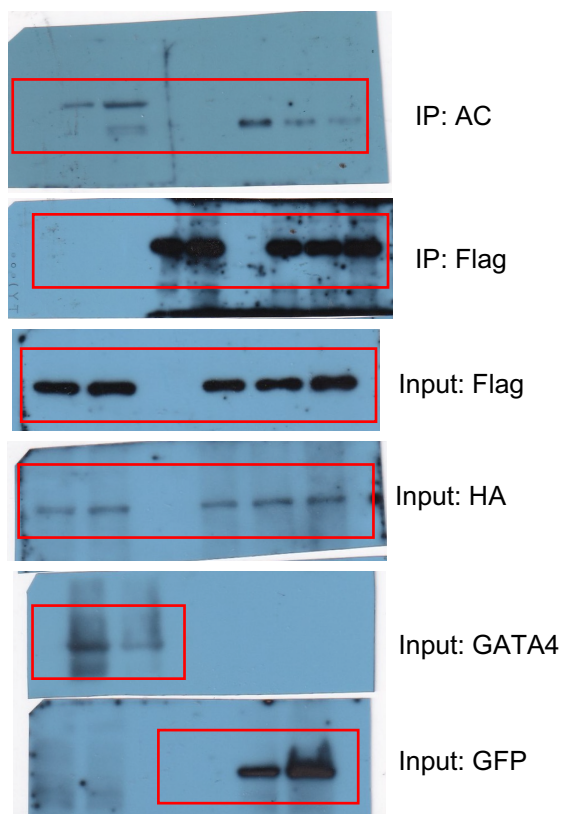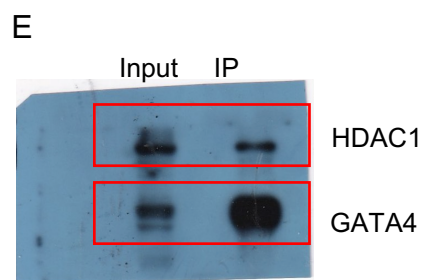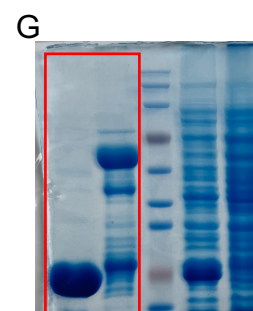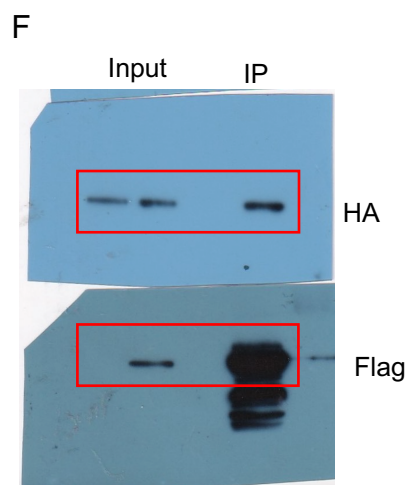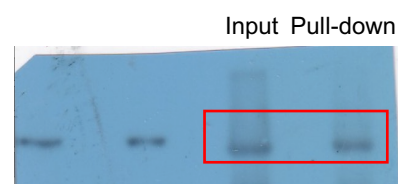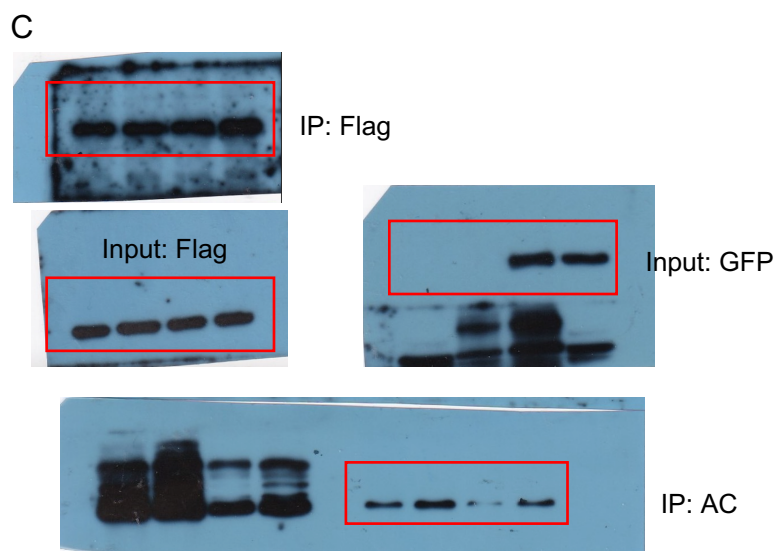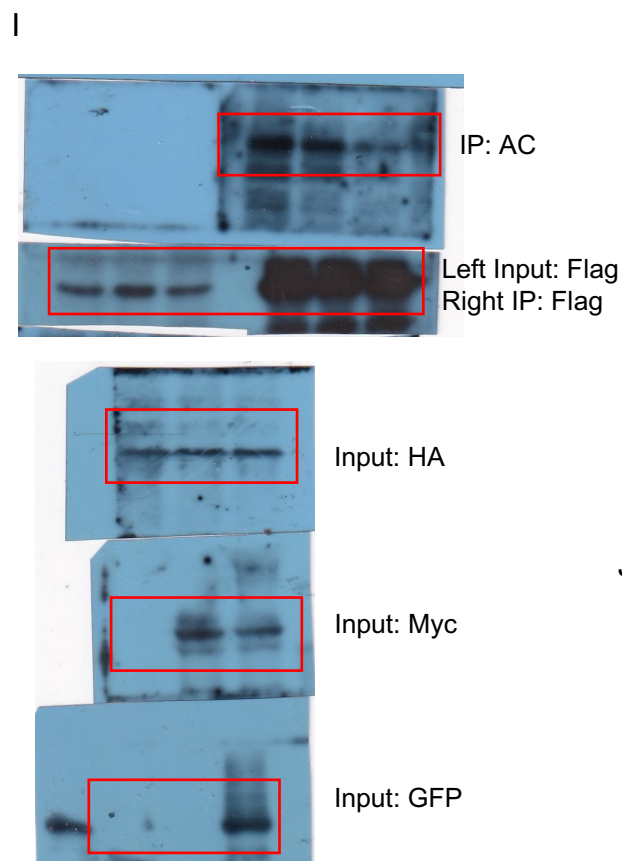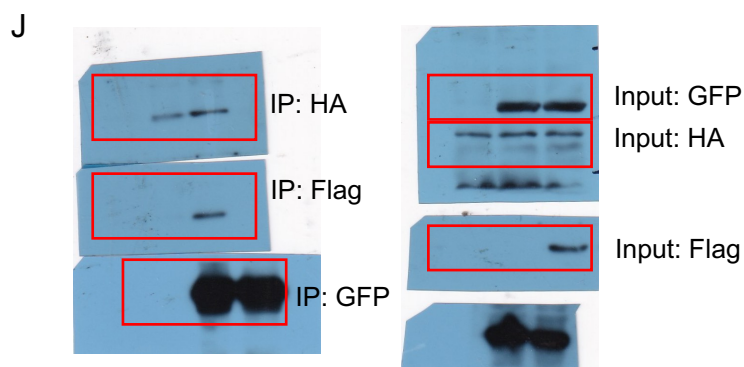

Figure 7

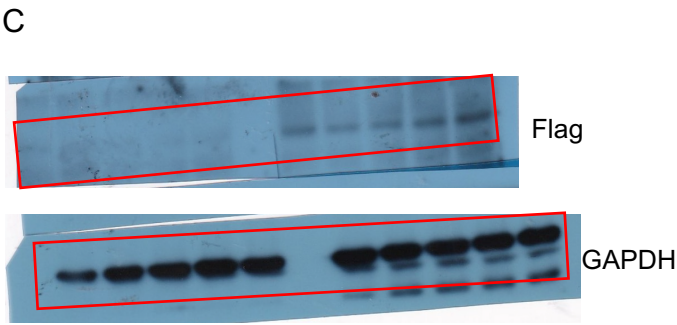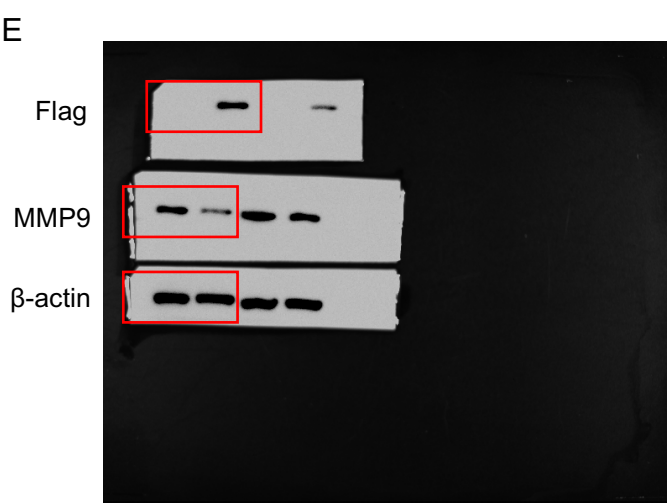

Supplement: Supplementary file 9 — Original WB data [file 41419_2024_6656_MOESM9_ESM.pdf]
