# Supplementary material for: GATA4 regulates the transcription of MMP9 to suppress the invasion and migration of breast cancer cells via HDAC1-mediated p65 deacetylation
Source: Cell Death Dis. 2024 Apr 23;15(4):289. doi: 10.1038/s41419-024-06656-z (PMC11039647; doi:10.1038/s41419-024-06656-z)
Supplement: Supplementary file 8 — Supplementary Figures and tables legends [file 41419_2024_6656_MOESM8_ESM.docx]

**Supplementary Figure 1**

1. GATA4 expression in TCGA tumors and adjacent normal tissues.

(<https://cistrome.shinyapps.io/timer/)>. This analysis showed that GATA4 expression was elevated in certain tumors, including breast cancer (BRCA), prostate adenocarcinoma (PRAD), and thyroid carcinoma (THCA), compared to their adjacent normal tissues

1. The expression data of GATA4 in breast cancer based on sample major subtypes were analyzed on UALCAN database. ([https://ualcan.path.uab.edu/index.html)](http://ualcan.path.uab.edu/)).
2. The relationship between GATA4 expression level and prognosis: overall survival (OS, up) and progression free survival (PFS, down)（high level: n=457; low level: n=453）

**Supplementary Figure 2**

1. Scratch wound-healing assay showed the effect of GATA4 overexpression in MDA-MB-231 cells.
2. Scratch wound-healing assay showed the effect of GATA4 knockdown in HCC1187 cells.
3. Transwell assay showed the effect of GATA4 overexpression in MDA-MB-231 cells on migration (up) and invasion (down).
4. Transwell assay showed the effect of GATA4 knockdown in HCC1187 cells on migration (up) and invasion (down).
5. Data of MDA-MB-231 cells are the means ± SDs from three determinations. **p* < 0.05; ***p* < 0.01.
6. Data of HCC1187 cells are the means ± SDs from three determinations. **p* < 0.05; ***p* < 0.01.

**Supplementary Figure 3**

1. Schematic illustration of mutated constitutions of MMP9 promoter.
2. The Pearson’s correlation and overlap coefficient are shown in bar graph format from at least 3 cells were analyzed (error bars, SEM). (Data of 5F).
3. The Pearson’s correlation and overlap coefficient are shown in bar graph format from at least 3 cells were analyzed (error bars, SEM). (Data of 6H).
4. Real-time PCR showing the effects of GATA4 overexpression on the mRNA expression levels of *MMP9*, *VEGFA*, *TNF-α*, and *uPA* in MCF7 cells. All experiments were repeated at least three times. Data are presented as means ± SD. **p* < 0.05, significant. **, *p* < 0.01.

**Supplementary Table 1**

The primers for shGATA4, ChIP, agarose gel electrophoresis, and plasmid amplifications.

**Supplementary Table 2**

The details of antibodies in our research.

**Supplementary Table 3**

The primers for quantitative real-time PCR.

**Supplementary Table 4**

The genes that positively associated with GATA4 in breast cancer (Pearson Correlation Coefficient > 0.1).
